# Virtual and CMC-Based Screening Identified Reticuline, an Intermediate of BIA Biosynthesis, as a Potential Agonist of D5R

**DOI:** 10.3390/molecules31081285

**Published:** 2026-04-14

**Authors:** Jing Mo, Zhihao Sun, Guoqing Xu, Guichun Zhang, Zhuangyuan Xie, Jinghao Zhao, Go Pei Heng, Zhaotong Cong, Liang Leng, Shilin Chen

**Affiliations:** 1 College of Pharmacy, Hubei University of Chinese Medicine, Wuhan 430065, China; 2 Innovative Institute of Chinese Medicine and Pharmacy, Chengdu University of Traditional Chinese Medicine, Chengdu 611137, China; 3 M Kandiah Faculty of Medicine and Health Sciences, Universiti Tunku Abdul Rahman, Kuala Lumpur 50744, Malaysia

**Keywords:** cell membrane chromatography, benzylisoquinoline alkaloids, reticuline, D5R, agonist

## Abstract

Natural products represent an important reservoir for GPCR ligand discovery. In this study, we established an integrated workflow combining virtual screening, biophysical validation, functional signaling assays, and transcriptomic profiling to identify reticuline, a dopamine-derived intermediate from the genus of *Stephania*, as a potential agonist of dopamine D5 receptor (D5R). Molecular docking revealed that most dopamine-derived compounds along the BIA synthetic pathway exhibit predicted binding affinities for the D5R that are lower than that of dopamine. As expected, the reticuline–D5R complex has a favorable predicted binding affinity of −7.9 kcal/mol. As for binding validation, direct interaction between reticuline and D5R was experimentally confirmed using cell membrane chromatography (CMC) and bio-layer interferometry (BLI), yielding a dissociation constant of 1.07 μM. cAMP assay demonstrated that reticuline activates D5R-mediated Gs-cAMP increasement in a concentration-responsive manner, which exhibits agonist-like activity with an EC_50_ value of 0.07 μM. The transcriptomic profiling further revealed that reticuline treatment induces transcriptional reprogramming in D5R-overexpressing cells, with enrichment of pathways related to ribosome biogenesis, mitochondrial oxidative phosphorylation, and neurodegenerative diseases. In summary, this study demonstrates that reticuline acts as a potential D5R agonist and highlights a systematic natural product-GPCR discovery strategy integrating computational prediction, experimental validation, and transcriptome-level mechanistic exploration.

## 1. Introduction

Natural products (NPs) and their analogs, biosynthesized by terrestrial and marine living organisms (plants, animals, insects, and microorganisms), represent a prolific source of structurally diverse pharmacophores [1,2]. Over the past century, systematic exploitation of natural active ingredients has led to their pivotal roles in prevention and treatment of various diseases, particularly in anti-cancer [3], metabolic disorders [4], and cardiovascular disease [5]. These active NPs and their derivatives exert pharmacological effects primarily through interactions with specific intracellular target proteins [6]. Consequently, the identification of target proteins represents a critical step in elucidating the mechanisms of NPs.

G protein-coupled receptors (GPCRs), characterized by a seven-transmembrane domain and comprising nearly 800 members in the human genome, represent one of the most intensively studied drug targets for various diseases. Approximately 36% of FDA-approved drugs target 121 GPCRs, exerting therapeutic effects by modulating downstream signaling pathways via heterotrimeric G proteins or β-arrestins [7]. NPs possess molecular architectures and bioactivities that differ from synthetic compounds, making them an important source for GPCR ligand discovery [8,9]. A literature analysis demonstrated that among the 643 unmodified natural products known to target GPCRs, a substantial majority (424 compounds) originated from plants [10].

As prototypical class A GPCRs of the central nervous system, dopamine receptors are implicated in numerous diseases and disorders, such as Parkinson’s disease, schizophrenia, and drug addictions [11,12]. Dopamine receptors consistently rank among the top 10 most intensively studied GPCR targets in drug development [13], and can recognize dopamine, the endogenous catecholamine. Dopamine can either stimulate cAMP production via Gαs proteins (D1-Like group, including D1R and D5R) or attenuate cAMP production by activating Gαi/o proteins (D2-like group, including D2R, D3R, and D4R) [14,15]. In recent years, an increasing number of natural small-molecule compounds have been reported to interact with dopamine receptors [16,17]. However, their specific mechanisms of action and targets have not yet been fully elucidated. As a widely existing substance in living organisms, dopamine also plays an important role in plants. In our previous research, we explored the dopamine synthetic pathway through a series of enzyme-catalyzed reactions to generate cepharanthine and other benzylisoquinoline alkaloids (BIAs) in the genus of *Stephania* [18,19]. Intriguingly, natural products harboring scaffolds such as benzyltetrahydroisoquinoline and tetrahydroberberine have been reported as ligands for dopamine receptors [16].

In recent years, substantial methodological advances have greatly accelerated GPCR ligand discovery. Advanced cell-based screening platforms, such as genome-wide pan-GPCR expression libraries, PRESTO-Tango and PRESTO-salsa systems, and CRISPRa/i-mediated modulation technologies, have substantially expanded the capacity for ligand screening of GPCRs [20,21]. In parallel, complementary affinity-based approaches, including cell membrane chromatography (CMC) and affinity mass spectrometry [8], have emerged as powerful tools for GPCR ligand discovery. Notably, CMC technology offers a unique affinity chromatography approach by utilizing GPCR-immobilized cell membranes as stationary phases, thereby exhibiting prominent advantages in components screening and receptor–ligand interaction identification [22]. Following primary screening, functional assays including the GloSensor™ cAMP assay, bioluminescence resonance energy transfer (BRET), Tango assay, and NanoLuc binary interaction technology (NanoBit) systems are routinely applied to characterize downstream signaling activity and pathway preferences of GPCR ligands [23,24]. In parallel with experimental screening, structure-based molecular docking has gained increasing prominence. With advances in protein structure determination, the activated conformations of the dopamine receptor family have been reported, providing a crucial foundation for structure-based drug screening [14,15]. These approaches provide significant insight into ligand–receptor screening and their interactions.

In this study, an integrated strategy combining virtual screening with experimental validation was employed to identify reticuline, an intermediate in BIA biosynthesis, as a potential D5R agonist. Based on molecular docking, CMC screening experiments assessed whether BIAs could interact with the receptor. The binding affinity between reticuline and D5R was detected using biolayer interferometry (BLI). Subsequently, functional activation of D5R was further examined via cAMP signaling assays, confirming that reticuline activates downstream cAMP accumulation mediated by the D5R. Furthermore, transcriptomic analysis revealed characteristic transcriptional changes induced by reticuline through D5R activation. This study thus provides a significant methodological reference for studies on target ligands for D5R and other GPCRs.

## 2. Results

### 2.1. Virtual Docking Identifies BIAs as Potential D5R-Interacting Compounds

**Figure 1:**
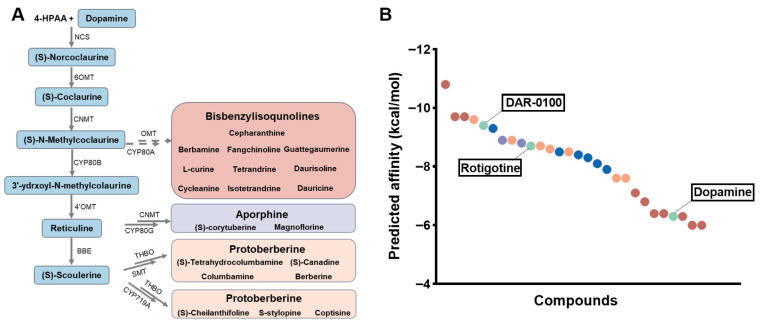
Candidate BIA compounds bind with D5R. (**A**) Proposed biosynthetic pathway for BIAs in *Stephania* species. (**B**) The predicted affinity of 28 compounds (DAR-0100, rotigotine, and dopamine, known D5R agonists, are included) and D5R via virtual molecular docking (green—positive agonist; blue-intermediates; red—bisbenszylisoqunolines; yellow—protoberberine; and purple—aporphine).

Based on the proposed biosynthetic pathway of BIAs in the genus of *Stephania* (Figure 1A), twenty-six compounds representing dopamine-derived intermediates and downstream alkaloids were selected [18]. Subsequently, virtual docking screening was performed on the D5R and these compounds to assess the receptor–ligand binding affinity (Table S1). The docking analysis revealed that aporphine and protoberberine alkaloids exhibited favorable predicted binding affinities for D5R. Compared to dopamine, bisbenzyliquinolines (bis-BIAs) exhibited poorer affinity, except for guattegaumerine, daurisoline, and dauricine. Among these intermediates, many BIAs showed a notable predicted binding affinity to D5R with corresponding binding energies below −7.0 kcal/mol (Figure 1B). This predicted affinity was comparable to or better than that of dopamine. Collectively, these results suggest that BIAs in the genus *Stephania* are potential D5R-interacting compounds.

### 2.2. Reticuline Binds to D5R via CMC and BLI

**Figure 2:**
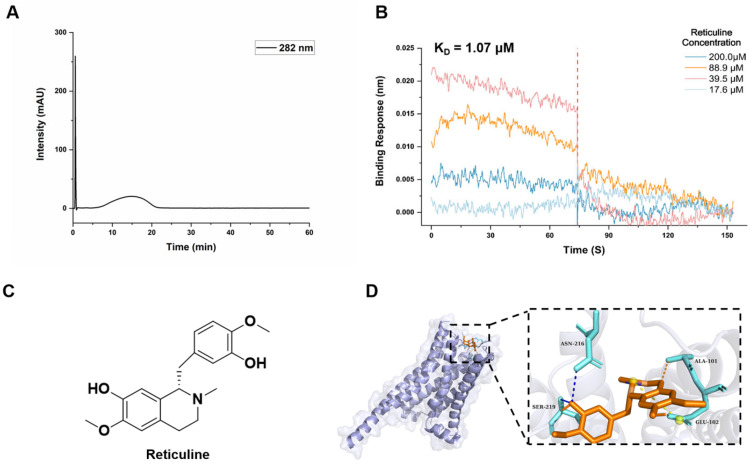
CMC and BLI identified that reticuline binds to D5R. (**A**) Chromatogram obtained by analyzing reticuline on a D5R-FLAG-tag/CMC system. (**B**) Real-time kinetic binding parameter of reticuline interacting with D5R based on BLI. (**C**) Chemical structure of reticuline. (**D**) Molecular docking pattern of reticuline and D5R (PDB: 8IRV).

To further validate the interaction between BIAs and the D5R receptor, cell membrane chromatography (CMC) was employed to experimentally assess their direct binding. Prior to these analyses, the expression of D5R fused with a FLAG tag was assessed at both the protein and mRNA levels in HEK293T cells. As expected, the expression of D5R-FLAG-tag was significantly higher in transfected cells compared with the vector control group (negative control transfected with an empty plasmid lacking the D5R coding sequence) (Figure S1A,B). Subsequently, a D5R-FLAG-tag/CMC model was constructed to identify candidate compounds binding with D5R. As depicted in Figure 2A, the interaction between reticuline and the D5R-FLAG-tag/CMC model yields a chromatogram with a distinct retention time at 10–20 min and exhibits a prominent absorption peak at 282 nm. As the other compounds were also tested, the known D5R antagonist berberine and agonist tetrahydrocolumbamine also show a prominent absorption peak, while other BIA retention signals were not detected (Figure S2). Reticuline serves as an important metabolic branch point capable of generating diverse alkaloid skeletons through C-C coupling reactions and subsequent structural modifications (Figure 2C) [18]. Further examination of the docking conformation indicated that reticuline could be stably accommodated within the ligand-binding pocket of D5R. The predicted binding mode revealed multiple stabilizing interactions with key amino acid residues, including hydrophobic interactions with ALA101, hydrogen bonds with ASN216 and SER219, and salt bridges with GLU102 (Figure 2D and Table S2). These interactions may contribute to the favorable binding profile observed for reticuline.

To further confirm the direct binding and quantitatively characterize the interaction, BLI analysis was performed. D5R protein fused with a Twin-Strep tag was first purified by immunoprecipitation using a FLAG antibody, and successful purification was confirmed (Figure S1C). The purified D5R protein was then immobilized on the biosensor surface, followed by exposure to reticuline at a range of concentrations. Reticuline induced concentration-dependent increases in the sensor response signal, demonstrating a direct interaction with D5R, with a dissociation constant (KD) at 1.07 μM (Figure 2B, Table S3). The CMC and BLI analysis results provide experimental evidence supporting a direct interaction between reticuline and D5R, thereby corroborating the computational predictions and identifying reticuline as a potential D5R-binding ligand.

### 2.3. Reticuline Activates D5R-Mediated cAMP Signaling

**Figure 3:**
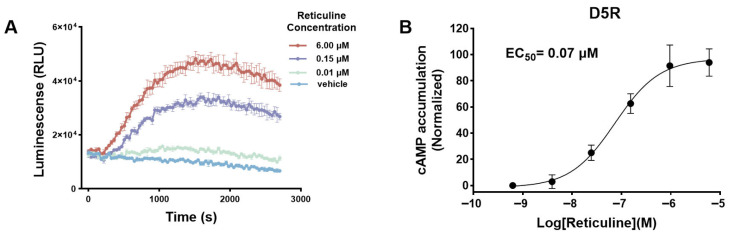
Reticuline exhibits D5R agonist activity to promote Gs-mediated cAMP accumulation. (**A**) Representative Glosensor^TM^ luminescence time-course data in one experiment over 45 min following application of reticuline to HEK293T cells expressing the D5R and cAMP probe Glosensor-22F. Data are presented as the mean ± standard error of mean (SEM) of three replicate measurements, expressed as relative light units (RLU) of luminescence. (**B**) Concentration–response curves of reticuline-induced cAMP accumulation. Data points represent the mean ± SEM of three independent experiments (each with three replicate wells).

To further evaluate the functional consequence of reticuline binding to D5R, a bioluminescence-based method was established for real-time monitoring of Gs-mediated intracellular cAMP production. This system facilitates dynamic detection of intracellular cAMP production within 45 min following ligand stimulation. HEK293T cells were seeded into a 96-well plate and transiently co-transfected with D5R and pGloSensor™-22F biosensor via Lipofectamine^TM^ 3000 transfection. After 24 h transfection, cells were treated with reticuline at increasing concentrations, while the known D5R agonist dihydrexidine (DAR-0100) was included as a positive control. Kinetic measurement revealed a characteristic increase in GloSensor™ luminescence intensity upon application of reticuline (Figure 3A), indicating activation of cAMP production. As shown in Figure 3B, reticuline stimulates cAMP formation in a concentration-response manner over a concentration range of 0.01 to 6 μM was clearly detected in live cells, with an EC_50_ value of 0.07 μM. Although the maximal luminescence response induced by reticuline exhibited a smaller magnitude of increase compared to DAR-0100 (Figure S3A,B), a clear and reproducible activation of cAMP signaling was consistently observed. This result suggests reticuline possesses D5R agonistic activity. In line with its activity at D5R, reticuline also activated the D1 receptor (D1R), indicating that it functionally active D1-like receptors and enhances cAMP signaling (Figure S4).

### 2.4. Transcriptome Profiling Reveals Dynamic Gene Expression Changes After Reticuline Treatment

**Figure 4:**
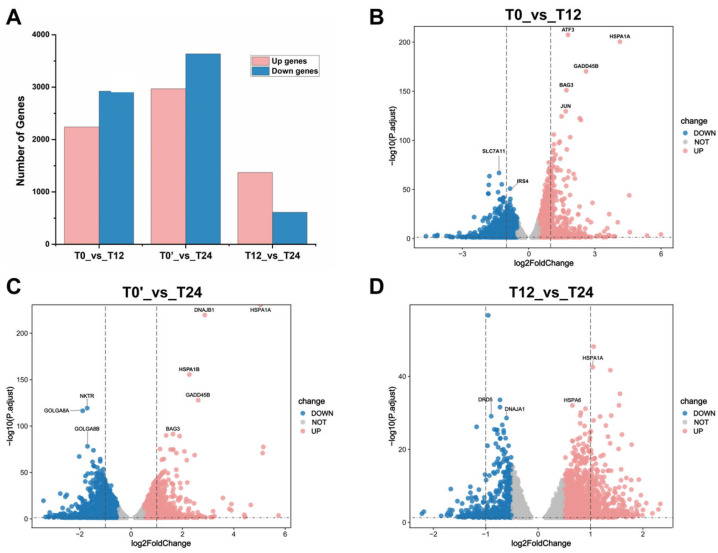
Transcriptome analysis comparing different groups. (**A**) The expression levels of DEGs in comparisons of different groups. (**B**–**D**) Volcano plot of transcriptional levels of DEGs in the three comparisons of T0_vs_T12, T0’_vs_T24, and T12_vs_T24, respectively.

**Figure 5:**
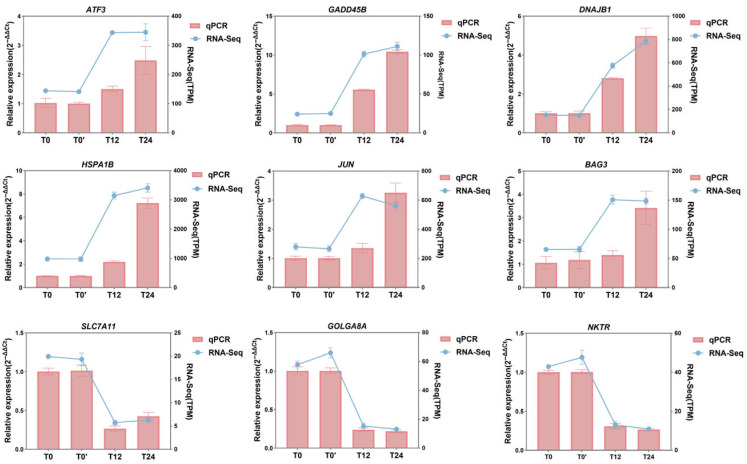
qPCR validation of selected genes. The columns represent the relative expression of qPCR (left *y*-axis) and the lines represent the TPM of RNA-Seq of key DEGs (adjusted *p* value ≤ 0.05).

To investigate the transcriptional responses induced by reticuline in D5R-overexpressing HEK293T cells, RNA sequencing was performed at 12 h (T12) and 24 h (T24) following treatment with 6 μM reticuline, with untreated cells serving as the baseline control (T0 and T0’). Unsupervised principal component analysis (PCA) revealed a clear separation between control and reticuline-treated samples, with distinct clustering observed among different treatment durations, highlighting a significant alteration in transcriptomic profiling (Figure S5). To identify genes exhibiting subtle but significant changes, the criteria for differential expression analysis were set at |log_2_FoldChange| ≥ 0.5 and an adjusted *p* value ≤ 0.05 [25,26]. In total, 4252, 5469, and 1793 DEGs were identified between the control group(T0) and 12 h treatment group, the control group (T0’) and 24 h treatment group, and the 12 h treatment group and 24 h treatment group, respectively (Figure 4A). Volcano plots analysis further illustrated the distribution of DEGs across comparisons using an adjusted *p* value ≤ 0.05 and |log_2_FoldChange| ≥ 0.5 as the criterion. The volcano plots revealed that, compared to control group, reticuline led to the upregulation of *HSPA1A*, *GADD45B*, and *BAG3*, while *SLC7A11*, *IRS4*, and *NKTR* showed significant downregulation (Figure 4B,C). The DEGs between T12 vs. T24 were also visualized, as shown in Figure 4D, with upregulation of *PCK2*, *HSPA1A*, *RIMS3*, and *HSPA6*, while downregulation of RNA-processing and signaling-related genes such as *HNRNPH1*, *SRSF1*, *GLS*, and *DNAJA1*. The results of the transcriptome data have been verified (Figure 5). While untreated D5R-overexpressing cells served as controls, inclusion of non-transfected (or mock transfected) HEK293T cells would further confirm the D5R specificity of observed transcriptional changes. The above results indicate that reticuline induces robust and time-dependent transcriptional reprogramming in D5R-overexpressing HEK293T cells.

### 2.5. Functional Enrichment Characteristics of DEGs

**Figure 6:**
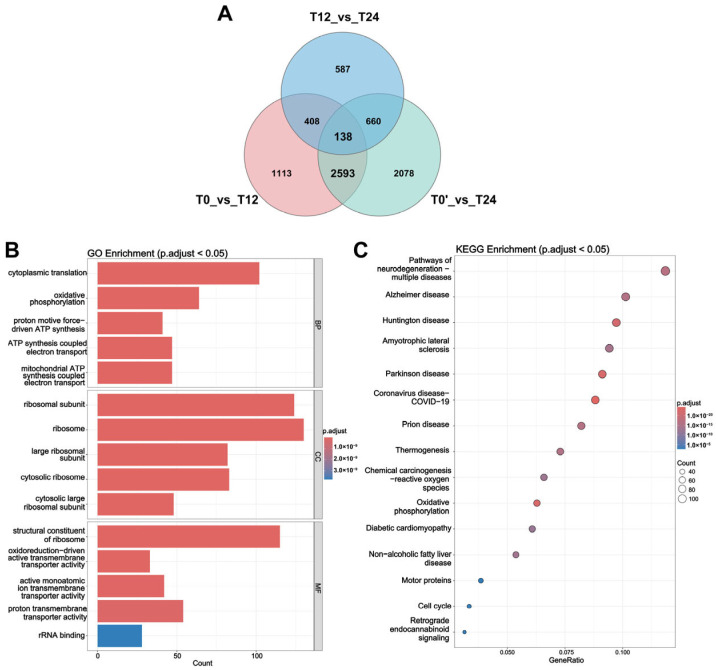
Functional enrichment analysis of differentially expressed genes (DEGs) across comparisons. (**A**) Venn diagram showing the common DEGs of T0_vs_T12, T0’_vs_T24, and T12_vs_T24. (**B**,**C**) GO and KEGG enrichment analysis of 2593 DEGs; these genes are shared by T0_vs_T12 and T0’_vs_T24, while excluding the overlapping part with T12_vs_T24 (see the Venn diagram). The bubble size represents the gene count, and the color scale indicates the adjusted *p* value ≤ 0.05. Only significantly enriched terms with adjusted *p* value ≤ 0.05 are displayed.

To identify core genes and pathways involved in the response to reticuline treatment, DEGs from the T0_vs_T12, T0’_vs_T24, and T12_vs_T24 comparisons were intersected. Venn diagram analysis identified 138 genes shared across the three comparisons, as well as 2731 overlapping genes common to both the T0_vs_T12 and T0’_vs_T24 comparisons (Figure 6A). GO and KEGG enrichment analysis of 2593 DEGs in the T0_vs_T12 and T0’_vs_T24 comparisons were performed to elucidate their functional implications. GO enrichment analysis revealed significant enrichment in cytoplasmic translation, while cellular component and molecular function categories were predominantly associated with ribosomal subunit and structural constituent of ribosome (Figure 6B). KEGG pathway analysis then consistently showed that these genes were predominantly enriched in the pathway of neurodegeneration-multiple disease, as well as oxidative phosphorylation, thermogenesis, and cell cycle pathways (Figure 6C). Supplementary analyses revealed that the T0_vs_T12 comparison exhibited enrichment signals and broad pathway coverage (Figure S6), whereas the T0’_vs_T24 and T12_vs_T24 comparisons were mainly associated with rRNA metabolic processes and dynamic regulation of mitochondrial function, respectively (Figures S7 and S8). The consistent overrepresentation of 47 genes involved in OXPHOS and mitochondrial function for both T12 and T24 may reflect a compensatory response to acute oxidative stress triggered by ligand stimulation (Table S5). Such a compensatory mechanism helps maintain mitochondrial homeostasis and cellular energy metabolism under mild stress conditions. The enrichment of neurodegenerative disease-related pathways further supports the close link between mitochondrial function and cellular stress responses. Taken together, these results suggest that reticuline treatment modulates transcriptional programs related to translational machinery, mitochondrial activity, and cellular metabolism.

## 3. Discussion

Biogenetically, BIAs are derived from dopamine, and a variety of natural and synthetic BIAs derivatives exhibit inherent dopaminergic activities [16]. Reticuline is positioned at the convergence of multiple alkaloid biosynthetic branches and can be further converted into a variety of pharmacologically active alkaloid scaffolds [27,28]. Virtual screening has emerged as a robust approach for uncovering novel GPCR chemotypes, with multiple investigations successfully identifying new ligands targeting D1R or D4R [29,30]. As the molecular docking results shown, multiple compounds within the BIA pathway of the genus *Stephania* exhibited favorable binding affinities towards D5R, with several compounds demonstrating predicted affinities superior to dopamine. Furthermore, when dopamine served as the benchmark, most bis-BIAs displayed poor affinity. Surprisingly, certain bis-BIAs demonstrated excellent binding potential, suggesting that these alkaloids also hold value during compound screening. Among the compounds, reticuline has been previously reported to competitively bind to the binding site of D1-like dopamine ligand ^3^H-SCH23390 in rats, suggesting its potential involvement in the modulation of dopaminergic signaling [31]. However, comprehensive experimental evidence demonstrating direct physical interactions between reticuline and dopamine receptors, as well as the functional outcomes of such interactions, remains insufficient.

Building upon these observations, this study provides experimental evidence confirming a direct interaction between some candidate compounds and the D5R receptor using CMC. As a result, reticuline shows a distinct retention profile (10–20 min at 282 nm), whereas other compounds with higher scores show no evidence of binding, suggesting that molecular docking scores may not fully correlate with actual screening outcomes. This may be related to the unreliability of molecular docking in predicting binding affinity, as binding energy prediction is affected by a variety of factors [32,33]. The reliability of virtual screening could be improved by incorporating optimization strategies, such as employing molecular dynamics simulations to assess the stability of the docking pose [34]. A number of docking programs have now been reported for drug discovery, particularly with the advancements in AlphaFold 3 and artificial intelligence algorithms, which have opened up new possibilities for high-throughput screening for protein structure prediction and accuracy [35,36,37,38]. In addition, BLI quantified the direct binding affinity between reticuline and D5R with a KD of 1.07 μM. This value aligns with previously reported affinities of reticuline for D1-like receptors (IC_50_ ≈ 1.8 μM) in rat striatal preparations [31], thereby establishing a robust biophysical foundation for reticuline as a D5R-binding ligand. Protein small-molecule interaction assays represent an effective technique for screening potential GPCR ligands [39]. As a high-throughput and highly stable method for screening potential active compounds, CMC has been employed to identify prospective ligands for GPCR proteins such as MrgX2 and H1R [22,40,41]. Current CMC methodologies are predominantly established for single target, and in our future work, we will construct multi-target cell membrane chromatography to validate the multiple dopamine receptors binding potential of compounds exhibiting favorable affinity in this study.

To determine whether this specific binding translates into biological activity, we characterized the effects of reticuline on the downstream signal transduction of D5R. Upon D5R receptor activation, it promotes the mediation of Gs protein and activates adenylate cyclase, thus increasing cAMP levels [17]. The results indicated that reticuline induces activation of the D5R-mediated Gs-cAMP signaling pathway with EC_50_ = 0.07 μM. The discrepancy between the KD and EC_50_ values may reflect receptor state, as GPCRs can adopt at least two conformational states: a G protein-coupled active state with high agonist affinity and an uncoupled inactive state with lower agonist affinity [42]. Based on receptor selectivity analysis results, reticuline may also activate D1R simultaneously. The maximal efficacy of reticuline is lower than that of the potent agonist DAR-0100, suggesting it may be a partial agonist. Partial agonists of D1-like dopamine receptors have been studied for the treatment of Parkinson’s disease (PD), and research into biased agonists of dopamine receptors holds greater significance for disease treatment [29,43]. To further investigate the signal selectivity of reticuline for D1-like receptors, a series of experiments including *β*-arrestin signalling, receptor internalization, and signal bias analysis will be conducted.

To further elucidate the signaling consequences of reticuline treatment following D5R engagement, we employed transcriptomic profiling to identify responsive genes and signaling pathways influenced by reticuline–D5R interaction. RNA-seq analysis revealed that reticuline treatment induces a coordinated remodeling of specific gene expression programs. Specifically, we observed the upregulation of a set of genes in the KEGG pathway of neurodegeneration-multiple disease, including *ATF3*, *HSPA1A*, *HSPA1B*, *GADD45B*, *BAG3*, *JUN*, and *DNAJB*, whereas genes *SLC7A11*, *IRS4*, *NKTR*, *GOLGA8A*, and *GOLGA8B* were significantly downregulated [44,45,46,47]. When comparing reticuline treatment and control groups, the GO enrichment analysis showed enriched pathways predominantly involved ribosome biogenesis, mitochondrial oxidative phosphorylation, and related molecular networks, while significant enrichment of genes associated with neurodegenerative diseases was identified via KEGG analysis. Notably, our previous studies have demonstrated that heterologous overexpression of receptors alone can alter basal transcriptional profiles [48,49]. We incorporated multiple treatment time points into the experimental design, while enabled a more refined dissection of dynamic transcriptomic changes, thereby improving the resolution and accuracy in distinguishing drug-induced effects from receptor overexpression-related transcriptional alterations.

Overall, our study demonstrates that reticuline acts as a potential D5R agonist. In addition, transcriptomic analysis revealed that reticuline significantly modulates gene expression change. However, the study relies entirely on D5R overexpression in HEK293T cells, a system that is prone to non-physiological signaling artifacts. Additionally, it would be highly informative to evaluate the effect of reticuline on non-transfected (or mock-transfected) HEK293T cells, which would significantly confirm the D5R-specific nature of the observed response. In subsequent investigations, neuronal or disease-related cell models (such as dopaminergic neurons or neuroblastoma cell lines) should be constructed to better approximate physiological conditions, and to investigate the similarities and differences in pharmacological effects between reticuline and endogenous dopamine. Additionally, this study has only utilized reticuline as a potential D5R agonist; future studies should also focus on other candidate BIA compounds and receptors.

## 4. Materials and Methods

### 4.1. Cell Culture and Transfection

Human embryonic kidney 293T (HEK293T) cells were purchased from the National Collection of Authenticated Cell Cultures (Shanghai, China). Cells were cultured in Dulbecco’s modified Eagle’s medium (DMEM, Gibco, Carlsbad, CA, USA) supplemented with 10% fetal bovine serum (FBS, Gibco, Carlsbad, CA, USA) and 1% Penicillin–streptomycin (Gibco, Carlsbad, CA, USA) at 37 °C in a humidified incubator (Haier, Qingdao, China) containing 5% CO_2_. When the confluence of cells reached 85–90%, they were passaged into a new T75 flask for further cultivation or seeded into 96-well plates for subsequent assays. According to the manufacturer’s instructions, HEK293T cells were transfected using Lipofectamine^TM^ 3000 transfection reagent (Thermo Fisher Scientific, Waltham, MA, USA).

### 4.2. Molecular Docking-Based Virtual Screening

The 3D structure of the D5R (Protein Data Bank ID: 8IRV) was downloaded from the PDB database (https://www.rcsb.org/) and preprocessed using PyMOL (v3.1.3) to remove non-standard residues and water molecules. The potential binding sites of D5R were predicted using the ProteinsPlus online tool (https://proteins.plus/). These predicted sites were then cross-referenced with the known binding site of the positive agonist Rotigotine to select the most appropriate docking pocket. AutoDock Tools (v1.5.6) was then used to further optimize the protein structure by adding hydrogen atoms, calculating Gasteiger charges, and saving the file in PDBQT format. We systematically standardized 28 small-molecule compounds using the RDKit. Among these, 26 compounds (including dopamine) from the biosynthetic pathway of BIA from the genus of *Stephania*, while the remaining two are known agonists (DAR-0100 and Rotigotine) of D5R. Molecular objects were generated from SMILES strings, followed by salt removal and hydrogen addition to ensure correct charge states and geometric integrity. Subsequently, molecules underwent standardization, deprotonation, and tautomer normalization, under simulated physiological pH 7.4 conditions. Then, ETKDGv3 was employed to generate 20 three-dimensional conformations. Each conformation was optimized using the MMFF94 force field to minimize energy. The conformer with the lowest energy was selected and saved in SDF format for molecular docking input. These compounds were subsequently processed in AutoDock Tools (v1.5.6) to redefine rotatable bonds and saved as PDBQT format. Semi-flexible molecular docking simulations were performed using AutoDock Vina (v1.1.2). We set Vina’s energy_range = 4, exhaustiveness = 32, and num_modes = 20, while the docking parameters were Number of GA Runs = 100 and Population Size = 900 in Autodock. Finally, PLIP tools and PyMOL (v3.1.3) were used to visualize the best binding poses of the compound–protein complexes [50].

### 4.3. Construction of D5R Cell Membrane Chromatography Model

D5R-FLAG plasmid, incorporating a FLAG tag in the C-terminus of D5R, was constructed to over-express human D5R in the HEK293T cell line. Lentiviral particles were generated by co-transfecting lentiviral vectors and auxiliary packaging plasmids into HEK293T cells using Lipofectamine^TM^ 3000 transfection reagent (Thermo Fisher Scientific, Waltham, MA, USA) for 48 h. These particles were subsequently used to infect pre-prepared HEK293T cells for 8 h, after which the medium was replaced. Following 48 h incubation, the cells were cultured in medium containing 2 μg/mL puromycin (Beyotime, Shanghai, China) for one week. The surviving cells, named D5R-FLAG-tag cells, were then expanded in medium supplemented with 1 μg/mL puromycin. To access the expression levels of D5R mRNA and protein, Western blot and quantitative real-time polymerase chain reaction (qRT-PCR) were utilized, respectively.

The establishment of D5R-FLAG-tag/CMC model was modified according to previous work [51]. Firstly, the D5R-FLAG-tag cell membrane stationary phase (D5R-FLAG-tag/CMSP) was prepared. For this purpose, D5R-FLAG-tag cells were harvested using trypsin (Gibco, Carlsbad, CA, USA), and the cell membrane suspension was obtained following ultrasonication and centrifugation (12,000× *g*, 4 °C, 20 min). Next, 50 mg silica gel carriers (SiO_2_-NH_2_) (Acchorm, Beijing, China), which were modified with FLAG-tag-specific antibodies (Huabio, Hangzhou, China), were incubated with the cell membrane suspension at 37 °C for 1 h at an agitation speed of 1400 rpm. After centrifugation at 1000× *g* for 5 min, the pellet was collected and washed three times with stroke-physiological saline solution, producing the D5R-FLAG-tag/CMSP. Secondly, to establish the D5R-FLAG-tag/CMC model, the D5R-FLAG-tag/CMC column was first obtained by wet-packing the D5R-FLAG-tag/CMSP into a column (10 × 2.0 mm i.d.), and then the column was connected with a liquid chromatography system (Wooking, Shanghai, China). As for drug screening, ultrapure water was used as the mobile phase, a 10 μL volume of the 1 mg/mL drug solution dissolved in 80% methanol was injected into the chromatographic system, and the flow rate was set to 0.3 mL/min.

### 4.4. Bio-Layer Interferometry (BLI) Binding Kinetics Assay

The HEK293T cells were transiently transfected with D5R plasmids with N-terminal FLAG tag and C-terminal Twin-Strep tag to express D5R protein. The purification of D5R protein was performed using immunoprecipitation with Anti-FLAG M2 affinity gel (Sigma-Aldrich, St. Louis, MO, USA). Firstly, cells were harvested and lysed in Pierce™ GPCR Extraction and Stabilization Reagent (Thermo Fisher Scientific, Waltham, MA, USA). The lysate was incubated with Anti-FLAG M2 affinity gel overnight at 4 °C with gentle rotation. The mixture was washed eight times with lysis buffer, and protein was eluted with 3× FLAG peptide (Sangon, Shanghai, China). The eluted protein was subsequently analyzed by SDS-PAGE and Western blotting to confirm the purification efficiency.

The binding affinity of D5R protein with reticuline was determined using the GatorPrime biolayer interferometry system (Gator Bio, Palo Alto, CA, USA). All steps were performed at 30 °C, 1000 rpm. Briefly, SAS sensors (Gator Bio) were dipped into the D5R protein solutions for 30 min for loading and then dipped into various concentrations of compounds (0–200 μM) in PBS, 1% DMSO buffer for 80 s, which was followed by an 80 s buffer wash to facilitate the dissociation of molecules from the protein. Background binding controls used a duplicate set of sensors that were incubated in a buffer without proteins. All the data were analyzed via Gator Bio data analysis software (GatorOne v2.7). The equilibrium dissociation constant (KD) values were calculated from the ratio of Koff to Kon, based on global fitting of several curves generated from serial dilutions of reticuline.

### 4.5. cAMP Assay

Human Dopamine Receptor D5R was cloned into mammalian expression vector pcDNA3.1(+), and the cAMP Glosensor^TM^ (22F) biosensor was obtained from Promega (Madison, WI, USA). Briefly, HEK293T cells were plated in 96-well plates (3 × 10^4^ cells per well) and cultured for 24 h at 37 °C and 5% CO_2_. A transfection complex (one well) contained Opti-MEM (Gibco, Carlsbad, CA, USA) (20 μL), Lipofectamine™ 3000 Reagent (0.2 μL), P3000™ Reagent (0.2 μL), and plasmids (100 ng total DNA, including 50 ng D5R expression plasmid and 50 ng pGlosensor^TM^ cAMP plasmid). The mixture was then incubated at room temperature for 20 min, after which fresh medium was added to bring the total volume to 100 μL. Cells were incubated in 100 μL transfection mixture for 24 h before the cAMP assay.

The cAMP accumulation was measured as previously described [52]. After 2 h incubation with replacement with 80 μL/well D-luciferin medium (Abcam, Cambridge, UK) at room temperature in the dark, 10 μL/well of IBMX solution (Sigma-Aldrich, St. Louis, MO, USA) was added to a final concentration of 200 μM. The plate was then maintained at room temperature for 20 min to inhibit cAMP degradation. For agonist studies, 10 μL/well reticuline (10 × final concentration) or HEPES-buffered MEM (vehicle control) was added. Luminescence was measured immediately using the SpectraMax iD3 multi-mode microplate reader. Log(agonist) versus dose–response curves and EC_50_ values were analyzed using GraphPad Prism 10.4.

### 4.6. Cell Treatment and RNA-Seq Analysis

HEK293T cells were transiently transfected with D5R recombinant plasmids constructed based on pcDNA3.1(+) vector. D5R-overexpressing HEK293T cells were treated with 6 μM reticuline for 12 h or 24 h, respectively, and then harvested for total RNA extraction. The RNA concentration and purity of total RNA were evaluated using a NanoDrop One spectrophotometer (NanoDrop Technologies, Wilmington, DE, USA) and Qubit 3.0 Fluorometer (Life Technologies, Carlsbad, CA, USA). RNA library preparation and high-throughput sequencing were performed according to Wuhan Benagen Technology Company’s instructions. For data analysis, raw sequencing reads were first quantified using kallisto to obtain gene-level expression estimates. Principal component analysis (PCA) was conducted based on the whole-genome expression profile to evaluate the overall separation among experimental groups. Differentially expressed genes (DEGs) were identified with DESeq2. Functional annotation of DEGs was performed through GO (Gene Ontology) and KEGG (Kyoto Encyclopedia of Genes and Genomes) enrichment analyses were performed using clusterProfiler (v4.8.0) in R (v4.3.1).

### 4.7. Western Blot

Total proteins were isolated from cell samples using cell lysis buffer. After centrifugation, protein concentrations were determined with the BCA protein assay kit (Thermo Fisher Scientific, Waltham, MA, USA). Equal amounts of proteins were separated by SDS-PAGE and subsequently transferred onto polyvinylidene difluoride (PVDF) membranes (Sigma-Aldrich, St. Louis, MO, USA). The membranes were blocked with 5% non-fat milk at room temperature for 1 h and then incubated overnight at 4 °C with primary antibodies, including FLAG M2 mouse monoclonal antibody (Sigma-Aldrich, St. Louis, MO, USA) or *β*-actin antibody (Abcam, Cambridge, UK). After washing three times with TBST (Servicebio, Wuhan, China), the membranes were incubated with the secondary antibody (Abcam, Cambridge, UK) at room temperature for 1 h. Following additional washes with TBST, protein signals were finally visualized in Tanon 5200 multi chemiluminescence imaging system (Tanon, Shanghai, China) with enhanced chemiluminescence (ECL) substrate (Bgbiotech, Chongqing, China).

### 4.8. Quantitative Real-Time PCR Verification Analysis

Total RNA extracted from the experimental samples was reverse-transcribed into cDNA using a reverse transcription kit (Takara, Beijing, China). Quantitative real-time PCR (qPCR) was conducted using a SYBR Premix Ex Taq II kit (Takara, Beijing, China) to evaluate the level of expression of D5R and key DEGs. The primer sequences are shown in Table S4. Thermal cycling conditions used for qPCR were the same as those used in our previous study [46], and the relative expression of the genes was calculated via the 2^−ΔΔCt^ method.

## 5. Conclusions

In this study, we employed molecular docking-based virtual screening to investigate the interaction between reticuline and activated D5R, revealing a predicted binding affinity of –7.9 kcal/mol. The direct binding between reticuline and D5R was validated by CMC, which showed a distinct retention profile (10–20 min at 282 nm). Additionally, BLI was used to quantify this direct binding, yielding a dissociation constant (KD) of 1.07 μM. Functionally, we demonstrated that reticuline activates the D5R-mediated Gs-cAMP signaling pathway, exhibiting characteristics of a potential D5R agonist. Furthermore, transcriptomic analysis revealed that treatment with reticuline in D5R-overexpressing cells induces transcriptional reprogramming, characterized by upregulation of genes related to ribosome biogenesis and mitochondrial oxidative phosphorylation. Overall, this study presents an integrated strategy combining computational prediction, binding validation, functional confirmation, and transcriptome analysis. Using this strategy, we systematically demonstrate that reticuline, an intermediate of BIA biosynthesis, constitutes a potential D5R receptor agonist.

## Data Availability

The original contributions presented in this study are included in the article/Supplementary Materials. Further inquiries can be directed to the corresponding author.

## Supplementary Materials

The following supporting information can be downloaded at: https://www.mdpi.com/article/10.3390/molecules31081285/s1, Figure S1: Construction of D5R overexpression cell line for CMC and BLI. (A) Relative mRNA expression levels of D5R in vector and OE-D5R-FLAG (**** *p* < 0.0001). (B) The protein level of D5R-FLAG in cell lysates was detected via Western blot. (C) The D5R-FLAG protein purified for BLI (L-cell lysates and IP-immunoprecipitation). Figure S2: Chromatogram obtained by analyzing compounds on D5R-FLAG-tag/CMC system. (A) berberine, (B) tetrahydrocolumbamine, (C) magnoflorine, (D) daurisoline, (E) dauricine, (F) berbamine. Figure S3: DAR-0100 mediates increased cAMP accumulation after D5R activation. (A) Representative GlosensorTM luminescence time-course data in one experiment over 45 min following application of DAR-0100 to HEK293T cells expressing the D5R and cAMP probe Glosensor-22F. Data are mean ± standard error of mean (SEM) of three replicate measurements, expressed as relative light units (RLU) of luminescence. (B) Concentration–response curves of DAR-0100 induced cAMP accumulation. Data points represent the mean ± SEM of three independent experiments (each with three replicate wells). Figure S4: Reticuline exhibits D1R agonist activity to promote Gs-mediated cAMP accumulation. (A) Concentration–response curves of reticuline-induced cAMP accumulation. (B) Concentration–response curves of DAR-0100 induced cAMP accumulation. Data points represent the mean ± SEM from three independent experiments (each with three replicate wells). Figure S5: PCA analysis of different groups. Figure S6: Functional enrichment analysis of DEGs in D5R overexpressing HEK293T cells upon 12h reticuline treatment. GO enrichment analysis of DEGs between T0 samples and T12 samples (A) and KEGG enrichment analysis (B). Figure S7: Functional enrichment analysis of DEGs in D5R overexpressing HEK293T cells upon 24h reticuline treatment. GO enrichment analysis of DEGs between T0’ samples and T24samples (A) and KEGG enrichment analysis (B). Figure S8: Functional enrichment analysis of DEGs in D5R overexpressing HEK293T cells upon 12h and 24h reticuline treatment. GO enrichment analysis of DEGs between T12 samples and T24 samples (A) and KEGG enrichment analysis (B). Table S1: Predicted binding affinity of 28 compounds in molecular docking study. Table S2: Key interactions for reticuline and reference ligands with D5R. Table S3: Binding kinetic parameters of reticuline and D5R at different concentration. Table S4: Primer sequences for real-time PCR. Table S5: Genes involved in OXPHOS and mitochondrial function at both T0_vs_T12 and T0’_vs_T24.

## Abbreviations

The following abbreviations are used in this manuscript:

BIAs	Benzylisoquinoline alkaloids
BLI	Bio-layer interferometry
cAMP	Cyclic adenosine monophosphate
CMC	Cell membrane chromatography
CMSP	Cell membrane stationary phase
D1R	Dopamine D1 receptor
D2R	Dopamine D2 receptor
D3R	Dopamine D3 receptor
D4R	Dopamine D4 receptor
D5R	Dopamine D5 receptor
DEGs	Differentially expressed genes
EC_50_	Half maximal effective concentration
ECL	Enhanced chemiluminescence
FDA	Food and Drug Administration
GO	Gene Ontology
GPCRs	G protein-coupled receptors
H1R	Histamine H1 receptor
IC_50_	Half maximal inhibitory concentration
KD	Dissociation constant
KEGG	Kyoto Encyclopedia of Genes and Genomes
MrgX2	Mas-related G protein-coupled receptor member X2
NPs	Natural products
PCA	Principal component analysis
PD	Parkinson’s disease
qPCR	Real-time quantitative polymerase chain reaction
RLUs	Relative light units
