# Supplementary figures and images for: Virtual and CMC-Based Screening Identified Reticuline, an Intermediate of BIA Biosynthesis, as a Potential Agonist of D5R
Source: Molecules. 2026 Apr 14;31(8):1285. doi: 10.3390/molecules31081285 (PMC13119369; doi:10.3390/molecules31081285)

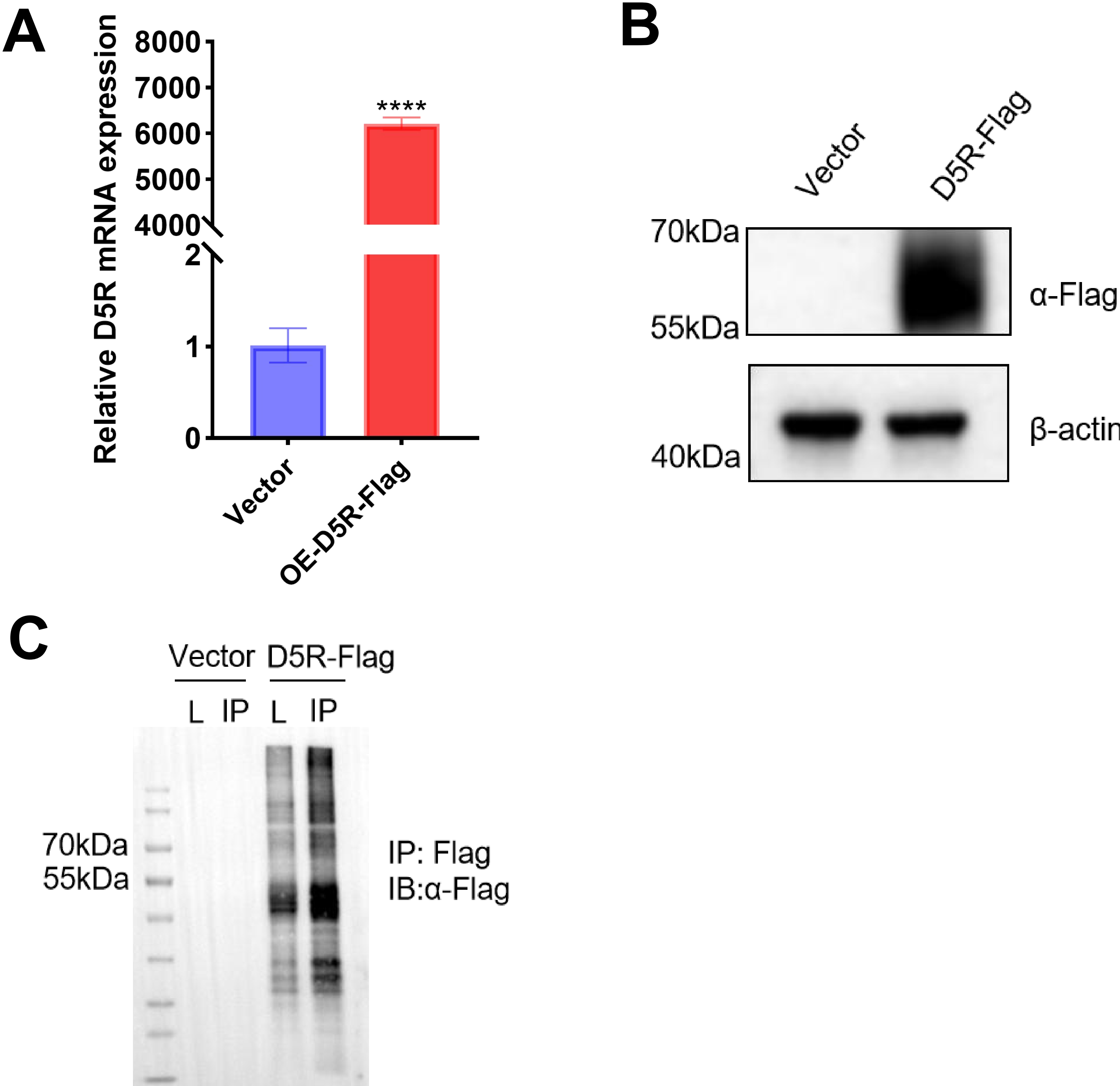

Supplement: Supplementary file 1 [file molecules-31-01285-s001.zip › Supplementary files/Figure S1.tif]

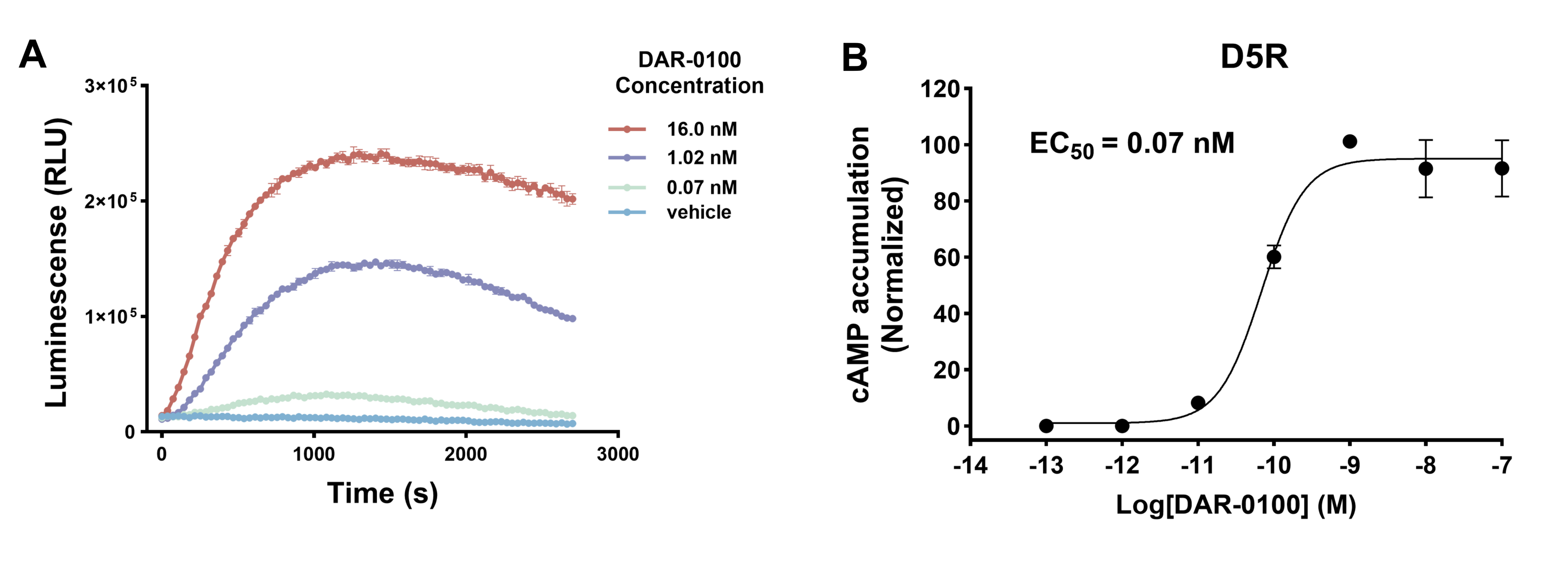

Supplement: Supplementary file 1 [file molecules-31-01285-s001.zip › Supplementary files/Figure S3.tif]

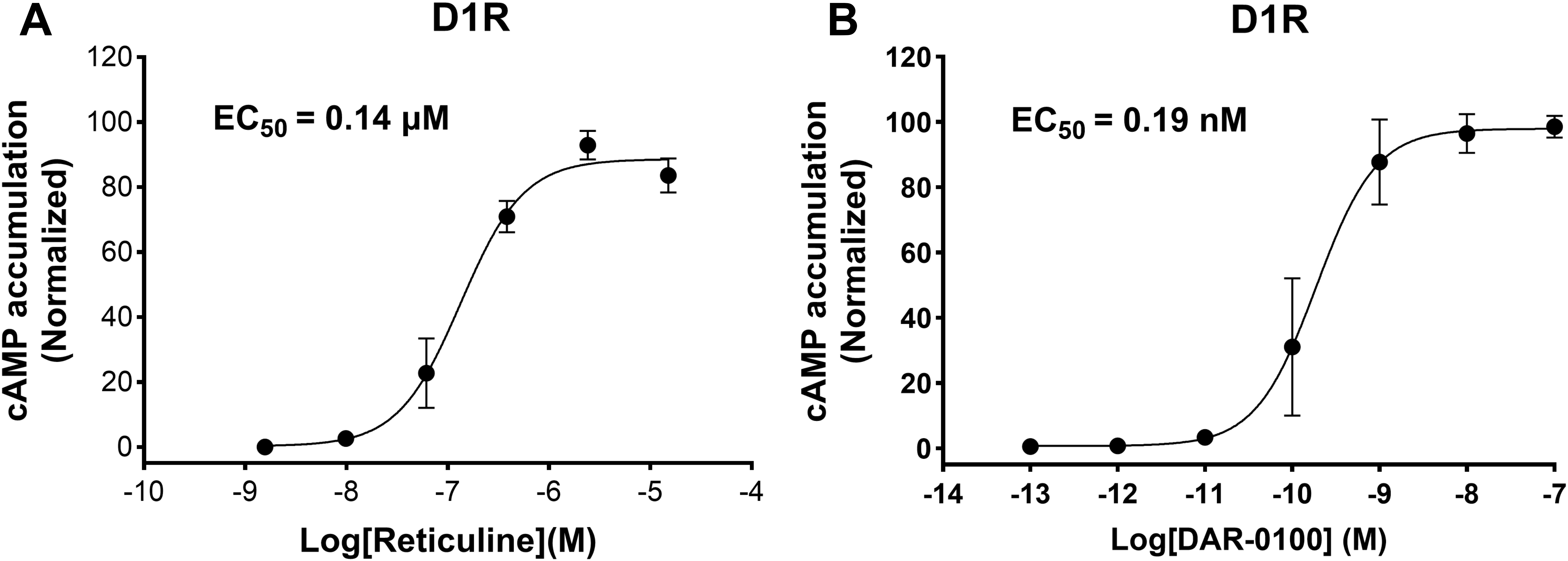

Supplement: Supplementary file 1 [file molecules-31-01285-s001.zip › Supplementary files/Figure S4.tif]
